# The win ratio in cardiology trials: lessons learnt, new developments, and wise future use

**DOI:** 10.1093/eurheartj/ehae647

**Published:** 2024-10-15

**Authors:** Stuart J Pocock, John Gregson, Timothy J Collier, Joao Pedro Ferreira, Gregg W Stone

**Affiliations:** Medical Statistics Department, London School of Hygiene and Tropical Medicine, London, WC1E 7HT, UK; Medical Statistics Department, London School of Hygiene and Tropical Medicine, London, WC1E 7HT, UK; Medical Statistics Department, London School of Hygiene and Tropical Medicine, London, WC1E 7HT, UK; Centre d'Investigations Cliniques Plurithématique 1433, INSERM, Université de Lorraine, Nancy, France; F-CRIN INI-CRCT (Cardiovascular and Renal Clinical Trialists), INSERM U1116, Centre Hospitalier Régional Universitaire de Nancy, Nancy, France; UnIC@RISE, Cardiovascular Research and Development Center, Department of Surgery and Physiology, Faculty of Medicine of the University of Porto, Porto, Portugal; Heart Failure Clinic, Internal Medicine Department, Unidade Local de Saúde de Gaia, Espinho, Portugal; Department of Medicine, The Zena and Michael A Wiener Cardiovascular Institute, Icahn School of Medicine at Mount Sinai, New York, NY, USA

**Keywords:** Clinical trial, Hierarchical composite outcome, Win ratio, Statistical methods, Presentation and interpretation, Systematic review

## Abstract

The win ratio method for analysing a composite clinical hierarchy of outcomes is growing in popularity especially in cardiovascular trials. This article gives a perspective on its use so far and the issues derived from that experience. Specifically, it focuses on the limitations of a conventional composite outcome; how does the win ratio work, what does it mean, and how to display its findings; guidance on choosing an appropriate clinical hierarchy of outcomes including clinical events, quantitative outcomes, and other options; the additional value of the win difference as a measure of absolute benefit: extension to stratified win ratio, subgroup analysis, matched win ratio, and covariate adjustment; determining trial size for a win ratio outcome; specific insights such as adaptive designs, use of repeat events, and use of margins and time averages for quantitative outcomes; a critique of potential misuses; availability of statistical software; and a statistical appendix on the methodological details. Throughout, each principle is illustrated by examples from specific cardiology trials. The article concludes with a set of recommendations for future use of the win ratio.

## Graphical Abstract

**Graphical Abstract:**
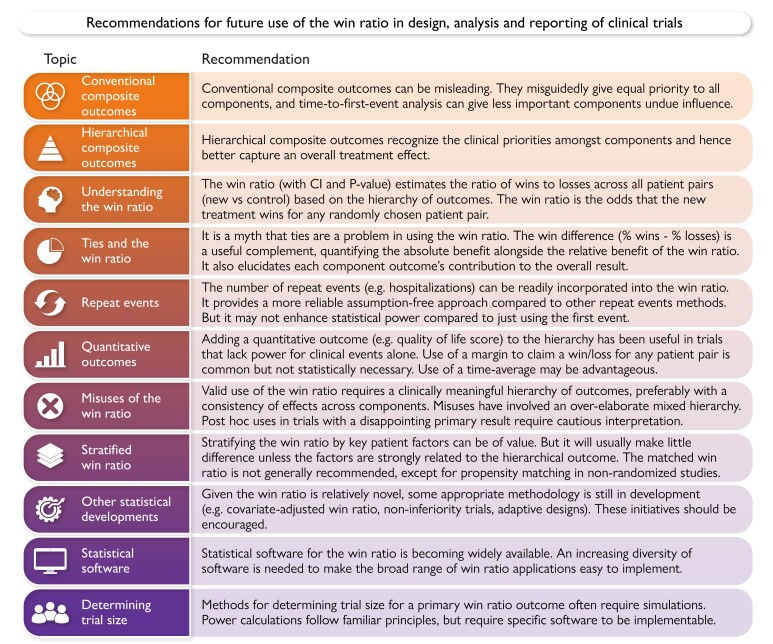
The graphical abstract figure explains the win ratio method, its advantages over conventional time-to-first event analysis of composite outcomes, and provides recommendations for future use of the win ratio in the design, analysis, and reporting of clinical trials. CI, confidence interval.

## Introduction

The win ratio, introduced in 2012,^1^ is an innovative approach to analysis of composite endpoints in randomized clinical trials (RCTs), its strength being to recognize the differing clinical importance of a composite’s components, prioritizing them in a clinical hierarchy. It can also incorporate repeat events, e.g. hospitalizations,^2^ and quantitative outcomes, e.g. quality of life scores.^3,4^

The win ratio method’s popularity has steadily grown, especially in cardiology trials so now is opportune time to review its role. In this article, we tackle several key issues:

1. The limitations of conventional composite outcomes
2. What the win ratio does and how to display and interpret it
3. Non-technical explanation of the estimate, confidence interval (CI), and *P*-value
4. Guidance on choosing an appropriate hierarchy of endpoints
5. Explanation of some complementary approaches, e.g. win difference
6. Determining trial size for a win ratio outcome
7. Availability of statistical software
8. Extensions to permit stratified, matched, or covariate-adjusted analyses
9. Specific insights regarding the use of repeat events and the use of margins and time averages for quantitative outcomes
10. Insights regarding misguided uses and misinterpretations of the win ratio
11. Conclusions including recommendations for future use of the win ratio

Our practical guidance is illustrated with real cardiology trial examples.

### The limitations of conventional composite outcomes

Many cardiology trials evaluate treatment efficacy by its impact on fatal and non-fatal events. Hence, the primary endpoint is often a composite of such events, e.g. in heart failure (HF), cardiovascular (CV) death or HF hospitalization, and in ischaemic heart disease, CV death, stroke, myocardial infarction, or revascularization.

Analysis commonly uses a proportional hazards model^5^ for time-to-first event, with hazard ratio, its 95% CI, and logrank *P*. Examples from the EMPEROR-Preserved^6^ and CLEAR^7^ trials are in *Table 1*. EMPEROR-Preserved is a typical pivotal trial in chronic HF. The time-to-first event is dominated by HF hospitalizations, so CV deaths that happen subsequently do not contribute to analysis. Also, this analysis ignores repeat hospitalizations.

**Table 1:** Two examples of conventional composite outcomes with an insight into their limitations

EMPEROR-Preserved trial^6^			
	Empagliflozin *N* = 2997	Placebo *N* = 2991	HR (95% CI)
Primary composite	415	511	.79 (.69–.90), *P* < .001
Components			Difference
HFH	259	352	−93
CV death	219	244	−25
Total HFH including repeats	407	541	−134
Problems with primary analysis			
All components of composite treated equally			
Ignores 148 (32%) CV deaths that occur after HF hospitalization			
Ignores 337 repeat HF hospitalizations (36% of the total)			

CLEAR trial^7^			
	Bempedoic acid *N* = 6992	Placebo *N* = 6998	HR (95% CI)
Primary composite	819	927	.79 (.69–.90), *P* < .001
Components			Difference
CV death	269	257	+12
Stroke	135	158	−23
Myocardial infarction	261	334	−73
Revascularization	435	529	−94
Problems with primary analysis			
No benefit in the most important component, CV death			
Greatest benefit in the least important component, revascularization			
Non-fatal components occur earlier and hence dominate the analysis			

*N*, number; HR, hazard ratio; CI, confidence interval; CV, cardiovascular; HF, heart failure.

The CLEAR trial illustrates the challenge in interpreting a four-component primary composite of CV events. The highly positive overall result is driven by benefits in coronary revascularization and myocardial infarction, the least clinically important components, while CV death has a slight numerical excess on bempedoic acid.

Thus, conventional composite endpoints do not directly take into account the fact that the component events may well vary in their clinical importance, e.g. deaths are more important than non-fatal events. While time-to-first event analyses often work well, they sometimes may fail to adequately represent a trial’s conclusions.^8^ A weighted combined effect measure could account for components of differing clinical relevance,^9^ but difficulties in agreeing a choice of weights, and consequent analytical complexities have inhibited its use. These limitations led to the development of the win ratio.

### The win ratio approach to a clinical hierarchy of endpoints

The win ratio is of value because most composite outcomes have a sensible hierarchy of components that reflects their clinical priorities. At its simplest, with two time-to-event outcomes of interest, death and hospitalization, the hierarchy is (i) death and (ii) hospitalization.

Such a hierarchy can be extended to more time-to-event outcomes, e.g. (i) death, (ii) stroke, (iii) myocardial infarction, and (iv) coronary revascularization. Alternatively, for repeat outcomes, one can replace time to event by number of events, e.g. a hierarchy (i) death and (ii) number of hospitalizations. In all these scenarios, deaths could be either all-cause or cause-specific, e.g. CV. Likewise hospitalizations could be all cause, CV, or condition specific, e.g. HF. Such choices depend on what best captures potential treatment effects.

In some RCTs, there are key quantitative outcomes such as change in self-assessed health status (e.g. KCCQ score), physical function (e.g. 6-min walking distance), or biomarkers (e.g. NT-proBNP).

**Figure 1:**
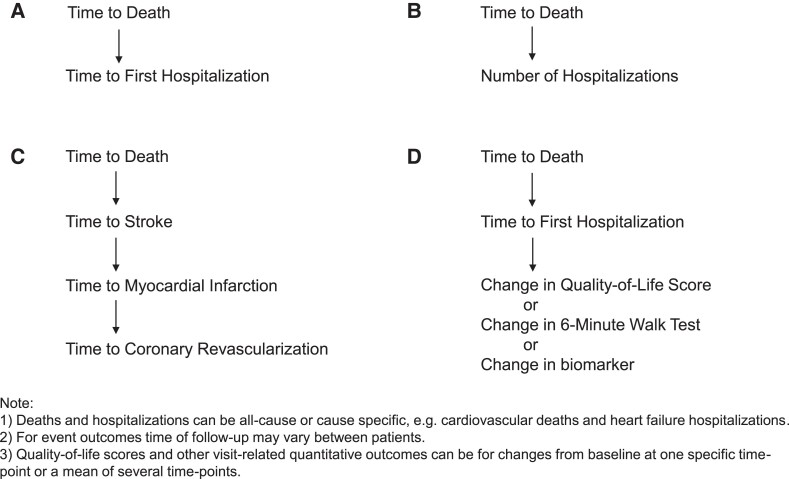
Four examples of hierarchical composite outcomes reflecting clinical priorities that have been used by randomized trials using the win ratio. (*A*) has two levels: time to death followed by time to first hospitalization; In (*B*), which also has two levels, time to first hospitalization has been replaced with number of hospitalizations. (*C*) has four levels; time to death, followed by time to stroke; then time to myocardial infarction; and finally time to coronary revascularization. (*D*) has three levels where the first two are time to events and the third level is a quantitative outcome, e.g. change from baseline in a 6-min walk test distance

*Figure 1* illustrates the types of clinical hierarchy that have been used. The choice of primary clinical hierarchy when designing a specific trial depends on several factors: what collection of outcomes best captures patient benefit and how extensive a hierarchy provides good statistical power given constraints on trial size.

Hierarchical composite outcomes are increasingly popular especially in cardiology trials. New statistical methods are needed to make them usable. The first innovation was Finkelstein and Schoenfeld’s^10^ non-parametric test that combined evidence from two or more hierarchical endpoints into a single *P*-value for a treatment difference (see Supplementary data online, *Appendix*). But one also requires an estimate of the magnitude of treatment effect and its 95% CI.

The win ratio was created for this purpose.^1^ The principle is as follows. Consider a RCT comparing new treatment vs. control with *N*_T_ and *N*_C_ patients, respectively. Then every patient on new treatment is compared with every patient on control, that is, *N*_T_ × *N*_C_ paired comparisons. Within each pair, one evaluates the hierarchical component outcomes in descending order of importance until one of the pair shows a better outcome than the other. If the patient on new treatment does better, it is called a ‘win’ whereas if the control patient does better, it is a ‘loss’.

Thus, among all paired comparisons, one accumulates a total of *N*_W_ wins; *N*_L_ losses and the rest are ties. The win ratio is *N*_W_/*N*_L_. This is sometimes called the unmatched win ratio and is consistent with the Finkelstein–Schoenfeld test.^10^ Obtaining the win ratio’s 95% CI involves a more complex calculation. Supplementary data online, *Appendix*, explains statistical details.

### The simplest hierarchy: death and a non-fatal event

**Figure 2:**
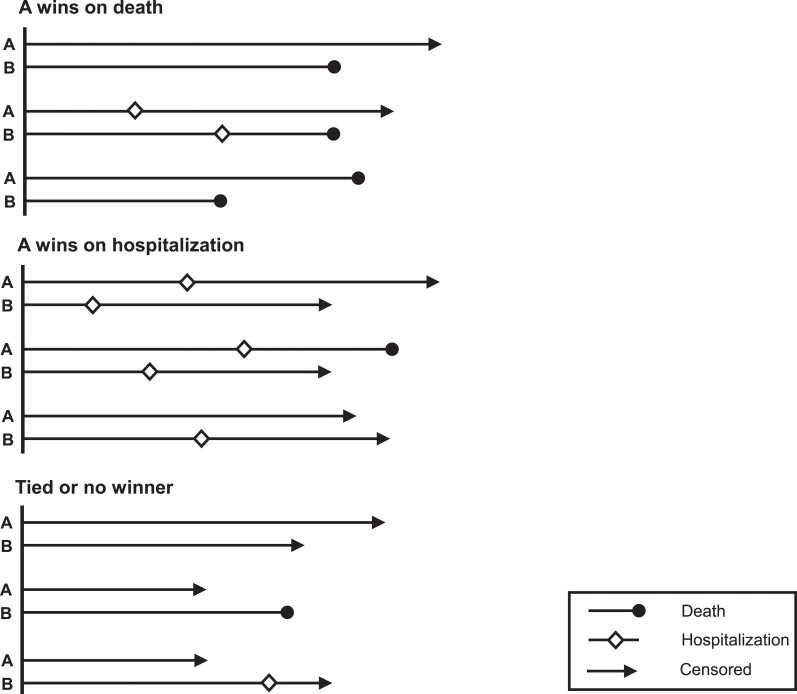
A schema showing nine patient pair (A, B) scenarios illustrating all the options that can arise for the simplest win ratio of the composite hierarchy of time to death and time to hospitalization. In the first set of three scenarios, A wins on the first level of the hierarchy, time to death, since B dies earlier and within the shared follow-up time. In the second set of three scenarios, A wins on the second level of the hierarchy, time to hospitalization. Note that although in the second of this set of scenarios A dies whereas B does not, the death occurs outside the shared follow-up time and is therefore ignored for this patient pair comparison. In the final set of scenarios, neither patient wins on either death or hospitalization and therefore they are tied

We now illustrate the simplest win ratio: a hierarchy comprising time to death and time to a non-fatal event, e.g. hospitalization. For each patient pair, we evaluate who ‘won’ over their shared follow-up time. If one or both patients died, then the one who lived longer is the ‘winner’. If neither patient died, but one or both were hospitalized, then the one who avoided hospitalization longer is the ‘winner’. If both patients survived without hospitalization over their shared follow-up time, it is a ‘tie’. *Figure 2* illustrates the possible options for any patient pair.

**Figure 3:**
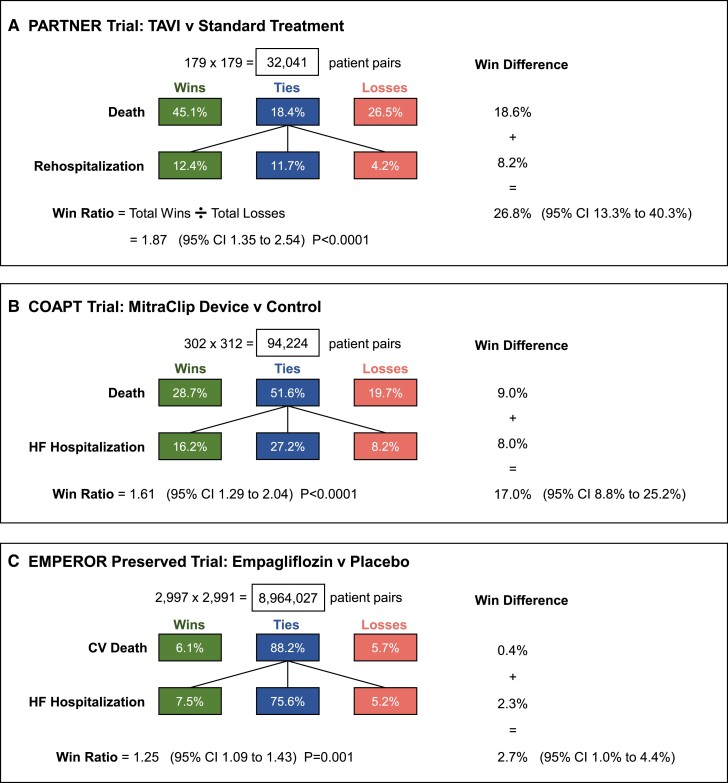
Three examples ((*A*) PARTNER,^11^ (*B*) COAPT,^12^ and (*C*) EMPEROR-Preserved^6^) of a two-level hierarchy win ratio analysis for a hierarchical composite of time to death and then time to a non-fatal event, hospitalization. Each panel shows the number of patients in each treatment group and the total number of patient pair comparisons. The green and red boxes show the percentage of all patient pair comparisons that were wins or losses, respectively, for the active arm. The blue boxes show the percentage of ties. The right-hand column gives win differences both overall and for each component. TAVI, transcatheter aortic valve implantation; CI, confidence interval

*Figure 3* shows three examples:

1. The PARTNER trial^11^ randomized patients with severe aortic stenosis not suitable for surgery to transcatheter aortic valve implantation (TAVI) or standard therapy. The primary endpoint was a hierarchy of time to death and time to rehospitalization. This was the first cardiology trial adopting the Finkelstein–Schoenfeld test. There were substantially more wins than losses both for deaths and hospitalizations leading to win ratio 1.87 (95% CI 1.35–2.54; *P* < .0001). In this high-risk population, incidence of ties (11.7%) was low.

How to interpret this win ratio? For any randomly chosen pair of patients who are not a tie, the estimated odds that the TAVI patient wins is 1.87. For such untied pairs, one can also estimate the probability that the TAVI patient wins, which is 1.87/(1.87 + 1) = .65.

1. The COAPT trial^12^ randomized patients with HF and mitral regurgitation to MitraClip device (*N* = 302) or medical therapy alone (*N* = 312). The primary endpoint, all HF hospitalizations within 2 years, had hazard ratio of .53 (95% CI .40–.70). Here, we analyse the hierarchical composite of time to death and then time to first HF hospitalization. Again, there were more wins than losses for both components resulting in win ratio 1.61 (95% CI 1.29–2.04; *P* < .0001).
2. The EMPEROR-Preserved trial^6^ randomized patients with preserved ejection fraction HF to empagliflozin (*N* = 2997) or placebo (*N* = 2991). The primary endpoint, a conventional composite of time to CV death or HF hospitalization over median 26.2 months, yielded hazard ratio of .79 (95% CI .69–.90; *P* < .001). Here, we analyse the hierarchical composite of time to CV death and time to first HF hospitalization. Both components had more wins than losses, much more so for hospitalizations. The consequent win ratio is 1.25 (95% CI 1.09–1.43; *P* = .001).

### Exploring the win ratio details, helped by the win difference

Like other outcomes, it is important to quantify absolute treatment effects, complementing the relative benefit of the win ratio. The win difference, also called net treatment benefit,^13^ is % wins − % losses. Calculating its 95% CI is more complex (see Supplementary data online, *Appendix*). We recommend reporting the win difference alongside the win ratio. It also helps to clarify each component’s contribution to the overall win ratio.

In *Figure 3*, the right-hand column gives win differences both overall and for each component. For PARTNER (*Figure 3A*), the win difference is 26.8%, indicating TAVI’s marked benefit in this high-risk population. This was mostly due to deaths, win difference of 18.6%, the win ratio for death alone being 1.70 (95% CI 1.23–2.38; *P* = .002). With a hierarchical composite rather than a conventional time-to-first composite, the impact on mortality was clearer and uncorrupted by any hospitalizations prior to death.

For COAPT (*Figure 3B*), the win difference is 17.0%, a substantial benefit evenly split between effects on death (9.0%) and on HF hospitalizations (8.0%).

For EMPEROR-Preserved (*Figure 3C*), the win difference of 1.9% is much smaller, as expected given the lower overall event rate: there were 75.6% ties, because most patients in both groups survived without HF hospitalization.

It is also useful to look at more conventional analyses of the composite and its components in a non-hierarchical fashion (see *Table 2*). For PARTNER, the reduction in risks of death and rehospitalization are clear and comparable. The conventional composite provides a highly significant combined effect, but fails to capture that 61 deaths (19 TAVI, 42 controls) occurred after a hospitalization. Only by doing a hierarchical composite do we capture all deaths in the win ratio analysis.

**Table 2:** Results for conventional composite endpoint and its components to relate to the hierarchical composite win ratio analyses of the three trials in *Figure 3*

PARTNER trial^11^	TAVI	Control	HR (95% CI)
No. of patients	179	179	
Death	55 (30.7%)	89 (50.7%)	.55 (.40, .74)
Rehospitalization	40	79	
Composite	76 (42.5%)	126 (71.6%)	.46 (.35, .59)

COAPT trial^12^	MitraClip	Control	
No. of patients	302	312	
Death	80 (29.1%)	121 (46.1%)	.62 (.48, .82)
HF hospitalization	92 (35.7%)	151 (56.7%)	.52 (.40, .67)
Composite	129 (45.7%)	191 (67.9%)	.57 (.45, .71)

EMPEROR-Preserved^6^	Empagliflozin	Placebo	
No. of patients	2997	2991	
CV death	219	244	.91 (.76, 1.09)
HF hospitalization	259	352	.71 (.60, .83)
Composite	415	511	.79 (.69, .90)

HR, hazard ratio; CI, confidence interval; TAVI, transcatheter aortic valve implantation; No., number; HF, heart failure.

For COAPT, a similar pattern emerges of strong device effects on both death and HF hospitalization. Again, the conventional composite ignores 124 deaths (43 MitraClip, 81 controls) that occur after HF hospitalization.

For EMPEROR-Preserved, the conventional composite and its components reveal what was also found with the win difference in *Figure 3*: that is, the treatment effect is driven by HF hospitalization with little effect on CV mortality. Note that 138 CV deaths (53 empagliflozin, 85 placebo) occurred after HF hospitalization and so are excluded from the non-hierarchical composite.

One expressed concern^14,15^ is that ties do not contribute to the win ratio. However, this issue is overcome by also presenting the win difference. That is, any measure of relative effect (win ratio, hazard ratio, or relative risk) should be accompanied by a measure of absolute effect.^16^

An alternative is to estimate the win odds^17^ = (% wins + $\frac{1}{2}$ ties) ÷ (% losses + $\frac{1}{2}$ ties). This is nearer unity, more so as the % ties gets bigger. But we feel it lacks insight and so is not recommended.

### Adapting the win ratio to include repeat events

In HF trials, there is interest in incorporating repeat hospitalizations into analysis either as a primary composite endpoint, e.g. PARAGON-HF^18^ or COAPT,^12^ or as a key secondary endpoint, e.g. EMPEROR^6,19^ or DELIVER.^20^ Sometimes death counts as an extra event, whereas others use a joint frailty model to adjust for its competing risk.^2^ In ischaemic heart disease, repeat events such as myocardial infarction and revascularization can be incorporated into secondary analyses, e.g. REDUCE-IT.^21^ Such analyses are complex and make strong assumptions and may not enhance statistical power.^2,22^

The win ratio can incorporate repeat events into the hierarchical composite endpoint. ATTR-ACT^23,24^ was first to do this. In evaluating tafamidis vs. placebo in 441 patients with transthyretin amyloid cardiomyopathy, the primary hierarchical composites were time to death and number of CV hospitalizations. The win ratio was 1.70 (95% CI 1.26–2.29; *P* < .0001), a more impressive result than achieved by alternative analyses. Note that this approach is assumption free.

In other trials, the use of repeat events in a win ratio may not enhance the treatment effect, e.g. *Table 3* for the EMPEROR-Reduced and EMPEROR-Preserved trials.^6,19^ For each trial, we converted the original primary endpoint into a hierarchical composite of time to CV death and time to first HF hospitalization. Both yielded highly significant win ratios of 1.340 and 1.251, respectively. Since CV deaths are prioritized in the win ratio and are less influenced by treatment than HF hospitalizations, *z*-scores are somewhat smaller, e.g. in EMPEROR-Reduced, *z* = 3.99 for win ratio compared with *z* = 4.41 in the Cox model.

**Table 3:** Two trial examples comparing three alternative ways of handling hospitalizations in the second level of a hierarchical composite: (i) time to first, (ii) no. of hospitalizations, or (iii) total days in hospital

Hierarchy	Win ratio (95% CI)	*z*-value^a^
EMPEROR-Reduced trial^19^		
CV death then first HFH	1.340 (1.160, 1.547)	3.99
CV death then no. of HFHs	1.335 (1.156, 1.541)	3.94
CV death then days in hospital due to HFH	1.330 (1.153, 1.134)	3.91
EMPEROR-Preserved trial^6^		
CV death then first HFH	1.251 (1.094, 1.430)	3.27
CV death then no. of HFHs	1.248 (1.091, 1.427	3.24
CV death then days in hospital due to HFH	1.238 (1.083, 1.416)	3.14

CI, confidence interval; CV, cardiovascular; HFH, heart failure hospitalization; no., number.

^a^The *z*-value is the standardized normal deviate corresponding to the *P*-value. For instance, *z* = 1.96, 2.58, 3.29, and 3.88 correspond to *P* = .05, .01, .001, and .0001, respectively.

Changing the second level of the hierarchy to be number of HF hospitalizations yields similar results, though for both trials, the consequent win ratio and its *z*-statistic become slightly smaller. Replacing the second level by number of days in hospital due to HF further reduces the win ratio estimates and *z*-scores.

This loss of statistical power when using repeat hospitalizations in HF trials^2^ appears surprising: e.g. in EMPEROR-Reduced and EMPEROR-Preserved, there were respectively 69 and 41 fewer repeat HF hospitalizations on empagliflozin compared with placebo on top of 104 and 93 fewer first HF hospitalizations respectively. Possible explanations are as follows: treatment effects may be more pronounced early on when first events dominate; repeat events have a highly skewed distribution with a few patients having many such events; and treatment switches may occur after a first event.^22^ However, there are trials, e.g. RELIEVE-HF,^25^ where recurrent hospitalizations are common and repeat event analyses enhance power.

Some trials, e.g. EMPULSE,^26,27^ have a hierarchical composite outcome containing both number of HF events and time to first event. But such an approach appears unnecessary: the win ratio finding is not enhanced and the result is harder to interpret.

### Extending the win ratio to quantitative outcomes

**Figure 4:**
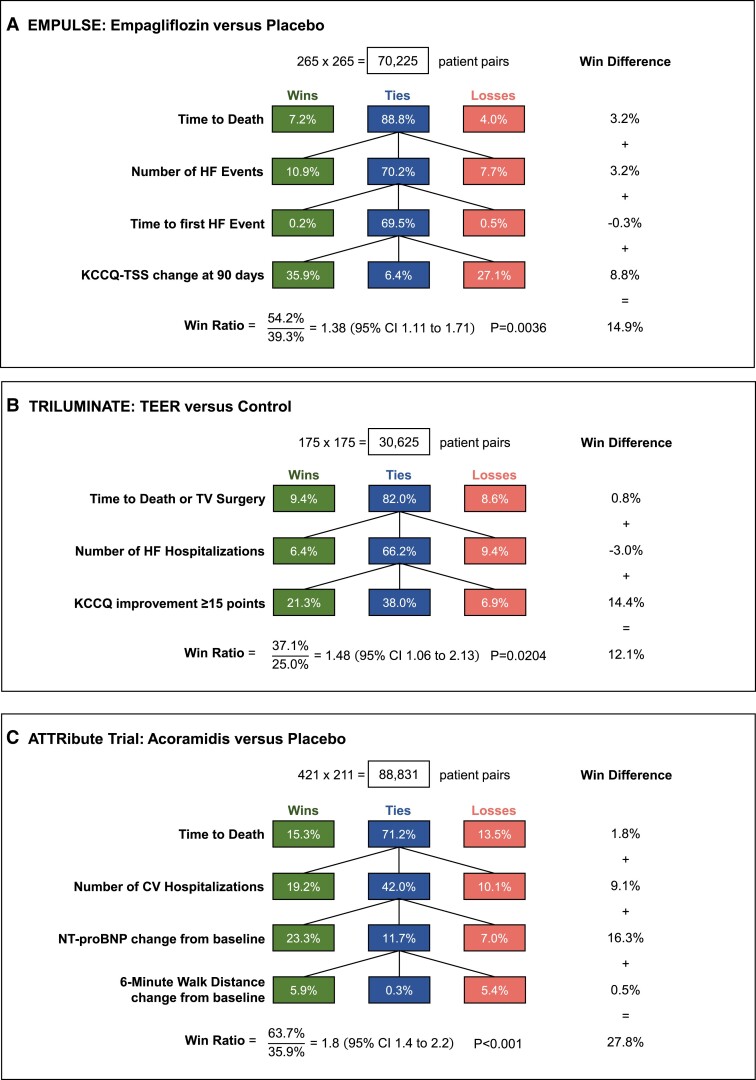
Examples of three trials (*A*) EMPULSE,^26,27^ (*B*) TRILUMINATE,^28^ and (*C*) ATTRibute-CM^29^ that extended the win ratio to include quantitative outcomes. Each panel shows the number of patients in each treatment group and the number of patient pair comparisons. The green and red boxes show the percentage of all patient pair comparisons that were wins or losses, respectively, for the active arm. The blue boxes show the percentage of ties. The right-hand column gives win differences both overall and for each component. HF, heart failure; KCCQ-TSS, Kansas City Cardiomyopathy Questionnaire-Total Symptom Score; CI, confidence interval; TEER, transcatheter edge-to-edge repair; TV, tricuspid valve

Another option is to extend the win ratio to incorporate outcomes other than clinical events, especially quantitative outcomes recorded at specific times.^3,4^ The clinical hierarchy includes not only deaths and non-fatal events but also other measures of patient well-being, measures of physical functioning, and surrogate biomarkers. There are two goals here: to achieve a more rounded view of treatment efficacy and to enhance statistical power. But such extended hierarchical outcomes need to be clinically meaningful and capture treatment benefits convincingly. There are also methodological challenges, illustrated by three examples (*Figure 4*).

The EMPULSE trial^26,27^ randomized 530 acute HF patients after initial stabilization to empagliflozin or placebo. The primary endpoint over 90 days was a hierarchical composite of time to death, number of HF events, time to first HF event, and change in KCCQ-Total Symptom Score (TSS) at 90 days. The consequent win ratio analysis is in *Figure 4A*. Note that 69.5% of patient pairs were tied after the first three hierarchy levels, since most patients on both treatments were alive and HF event free. Hence, the fourth level KCCQ-TSS change at 90 days contributed most to the positive result, win ratio of 1.38 (95% CI 1.11–1.71; *P* = .0036). Regarding absolute benefit, the overall win difference 14.9% was largely due to KCCQ-TSS change (win difference 8.8%) leaving only 6.4% tied.

There are two specific issues here: what were the rules defining win, tie, or loss for this quantitative outcome and how were missing data handled.

In EMPULSE, for any patient pair, a win (or loss) required the 90-day change in KCCQ-TSS to be ≥5 points better (or worse) for the empagliflozin patient compared with the placebo patient. It was a tie if the difference was <5 points. This fixed margin (5 points) is open to debate, and so sensitivity analyses used alternative criteria for a win: any difference, ≥2 points, ≥10 points, and ≥15 points. The estimated win ratio got slightly smaller as the margin got less, but so did the CI width, meaning the *P*-value scarcely changed.

It is debatable whether margins are necessary for valid use of the win ratio. The statistical argument against margins is that the inference is like other non-parametric tests in essentially ranking patients from worst outcome (first death after randomization) to best outcome (event free with the largest quantitative improvement), with the added complication of differing follow-up times. The clinical argument is that a win requires some clinically meaningful difference, a 5-point margin for KCCQ. We suspect the choice of margin, yes or no, will make little difference if the EMPULSE experience is generalizable.

The second key issue is how to account for missing data. If the quantitative outcome is measured multiple times, the latest time point with common shared data may be used. If data are entirely missing for a patient, then all their paired comparisons count as ties. Compared to the primary EMPULSE result in *Figure 4A*, frequency of ties increases from 6.1% to 18.7%. The consequent win ratio becomes 1.43 but with a wider 95% CI of 1.14–1.78. Instead, the pre-defined primary analysis used a multiple imputation algorithm.^27^ Analysis becomes more complex but is generally thought more appropriate.

Our second example, the TRILUMINATE trial,^28^ randomized 350 patients with tricuspid regurgitation to transcatheter edge-to-edge repair (TEER) or medical therapy (control). Over 1 year, the primary endpoint was the hierarchical composite of (i) time to death or tricuspid valve surgery, (ii) time to hospitalization, and (iii) improvement of ≥15 points in KCCQ score at 1 year (*Figure 4B*).

Note that the change in KCCQ score became binary, ≥15 points yes or no. Hence, for any patient pair, if both were yes (or both were no), then it is a tie. Only for those pairs with one yes and one no do we have a win or loss for TEER. Any dichotomizing of a quantitative outcome loses statistical power and insight into details of any treatment effect^30,31^ and hence is not recommended. Having said that, in TRILUMINATE, the treatment difference in KCCQ change was so marked that any analysis is highly significant. However, TRILUMINATE is open label so such a subjective outcome carries a risk of bias.

Another concern with TRILUMINATE is that the overall win ratio of 1.48 (95% CI 1.06–2.13; *P* = .0204) was driven by KCCQ with no evidence of a benefit on CV events, i.e. the win differences for the three levels of the hierarchy +.8%, −3.0%, and +14.4%, respectively, yield an overall win difference of 12.1%. Like a conventional composite outcome with disparate findings across components, the consequent overall summary, the win ratio, does not fully describe what happened. For TRILUMINATE, the TEER group clearly had a better self-assessed health status at 12 months, but to what extent did bias due to lack of blinding play a part?

Our third example, the ATTRibute-CM trial,^29^ randomized 632 patients with transthyretin amyloid cardiomyopathy in a 2:1 ratio to acoramidis hydrochloride or placebo. Over 30 months, the primary hierarchical composite outcome comprised death, number of CV hospitalizations, change in NT-proBNP, and change in 6-min walk distance. For these two quantitative components, any paired comparison used the last visit where both patients had assessments. The win/loss criterion had a margin of ≥500 pg/mL difference for NT-proBNP, whereas 6-min walk distance had no margin.

The win ratio for ATTRibute-CM was 1.8 (95% CI 1.4–2.2; *P* < .001) (*Figure 4C*). The overall win difference of 27.8% had its greatest contribution from change in NT-proBNP (win difference 16.3%) with number of CV hospitalizations also relevant (win difference 9.1%). With concern about relying on a biomarker change so much, sensitivity analyses were done with it removed. For death and CV hospitalization alone, the win ratio was 1.5 (95% CI 1.1–2.0), whereas adding in 6-min walk distance made little difference: win ratio 1.4 (95% CI 1.1–1.8).

ATTRibute-CM illustrates the uncertainties in defining what clinical hierarchy to choose as primary: during the trial’s course, the investigators twice changed their mind.

For these three examples, it is also informative to present conventional results for each separate component of the hierarchical composite (see *Table 4*) to complement the win ratio findings in *Figure 3*.

**Table 4:** Results for conventional analysis of each component to relate to the hierarchical composite win ratio analyses of the three trials in *Figure 4*

EMPULSE^26, 27^	Empagliflozin	Placebo	
No. of patients	265	265	
Deaths	11	22	
1+ HF event	28	39	
Composite	37	57	Hazard ratio .65 (95% CI .43, .99)
All HF events	36	52	
Mean baseline-adjusted 90-day KCCQ change	Difference +4.45 (95% CI .32, 8.59)		

TRILUMINATE^28^	TEER	Control	
No. of patients	175	175	
Death or TV surgery	16	18	
Annual rate of HF hospitalizations	.21	.17	
KCCQ improvement ≥15 points	73	39	
Mean KCCQ change at 1 year	Difference +11.7 (95% CI 6.8, 16.6)		

ATTRibute-CM^29^	Acoramidis	Placebo	
No. of patients	421	211	
Deaths	39	22	
CV hospitalizations	Rate ratio .496 (95% CI .355, .695)		
Geometric mean change in NT-proBNP	Ratio .529 (95% CI .463, .604)		
Mean change in 6-min walk test, m	Difference +39.6 (95% CI 21.1, 58.2)		

HR, hazard ratio; CI, confidence interval; KCCQ, Kansas City Cardiovascular Questionnaire; TV, tricuspid valve; TEER, transcatheter edge-to-edge repair.

For EMPULSE, there are fewer deaths and HF events on empagliflozin vs. placebo such that the conventional composite of time-to-first event was borderline significant: HR .65 (95% CI .43, .99). For KCCQ-TSS, the baseline-adjusted 90-day mean change had a modest treatment effect, though this simple analysis is somewhat flawed by ignoring the deaths. Overall, one can see how the consistent signal for deaths, HF events, and KCCQ combined to give clear evidence of treatment benefit.

For TRILUMINATE, the conventional analyses of each component of the hierarchical composite reinforce what the win difference showed in *Figure 4B*: the evidence of treatment benefit was confined to KCCQ improvement.

For ATTRibute-CM, the conventional analyses of each component (see *Table 4*) were compatible with the win differences in *Figure 4C*: treatment benefit being driven by fewer CV hospitalizations and a marked effect of NT-pro BNP. Acoramidis also improved 6-min walking distance, but this was less evident in the win ratio since it was last in the hierarchy with few ties remaining by then.

### Other uses of the win ratio

We now explore some innovative uses of the win ratio.

#### For an ordinal primary outcome

The SOS-AMI trial^32^ will assess the clinical efficacy of self-administered selatogrel vs. placebo when symptoms suggestive of recurrent acute myocardial infarction (AMI) occur. The primary outcome is an ordinal scale, the six outcomes ranked from worst to best being:

• Death within 7 days
• AMI with compromised electro-haemodynamics within 2 days
• ST-elevation myocardial infarction (STEMI) within 2 days
• High-risk non-ST-elevation myocardial infarction (NSTEMI) within 2 days
• NSTEMI with peak cardiac troponin ≥10 times normal within 2 days
• None of the above

Based on Wang and Pocock,^33^ the win ratio method will be applied to all patient pairs (selatogrel vs. placebo), the winner being the one with the lower rank. Many ties are anticipated since most patients will be event free. An alternative estimator for such an ordinal outcome is the common odds ratio,^34^ but this assumes the same improvement (odds ratio) applies to all five cut-offs in the ordinal scale. Hence, regulators encouraged the win ratio approach. Note that a conventional Mann–Whitney test is applicable to such an ordinal outcome, but does not elicit an estimate of treatment effect.

#### Adjusting a quantitative outcome for the competing risk of death

Many HF trials have quantitative outcomes, e.g. KCCQ score, recorded at several visits, with death being an informative censoring. Simple quantitative analyses assuming deaths missing at random can mislead. To overcome this, the DELIVER trial^20^ used a win ratio analysis for a hierarchical composite of death and KCCQ-TSS at 8 months, recognizing prior death as a worse outcome. The consequent win ratio of 1.11 (95% CI 1.03–1.21; *P* = .009) demonstrated that dapagliflozin improved HF symptoms, a valuable secondary finding.

The RECHARGE trials^35^ extend the same principle, a hierarchical composite of death and quality of life, into a longer-term evaluation of percutaneous coronary intervention (PCI) vs. coronary artery bypass graft (CABG) in female and ethnic minority patients with multi-vessel or left main coronary artery disease. The hierarchy comprises (i) time to death over 3–5 years and (ii) time-averaged change from baseline in the 12-Item Short Form Survey (SF-12) version 2 questionnaire, which is measured at 1, 3, 6, and 12 months and annually thereafter (with 5-point margin). The consequent win ratio captures the essence of a hierarchy of relevance to patients: (i) do I know who lived longer? and (ii) if not, who experienced the better quality of life? This bypasses many of the complexities and controversies in previous trials of PCI vs. CABG using conventional composites of death, myocardial infarction (MI), stroke, and revascularization.^36,37^

In general, for a quantitative outcome in the composite hierarchy, it is good practice to use an average value across several time points to better capture the overall effect.

#### Extending the clinical hierarchy to multiple outcomes

The DAPA-MI trial^38^ randomized 4017 MI patients without diabetes or HF to dapagliflozin or placebo with mean follow-up of 11.6 months. The original primary endpoint was CV death and HF hospitalization, but blinded interim data revealed low event rates and a lack of statistical power. Hence, a hierarchical primary composite outcome was defined with the following seven components: death, HF hospitalization, myocardial infarction, atrial fibrillation/flutter, type 2 diabetes, New York Heart Association (NYHA) class, and weight decrease ≥5%.

The first five steps determined win or loss for any patient pair using time to event, while win or loss for NYHA class and weight decrease used data from the last visit done for both patients. The consequent win ratio of 1.34 (95% CI 1.20, 1.50; *P* < .001) led to the conclusion that ‘there were significant improvements in cardiometabolic outcomes’. The original primary had hazard ratio of .95 (95% CI .64, 1.40), showing no effect.

**Figure 5:**
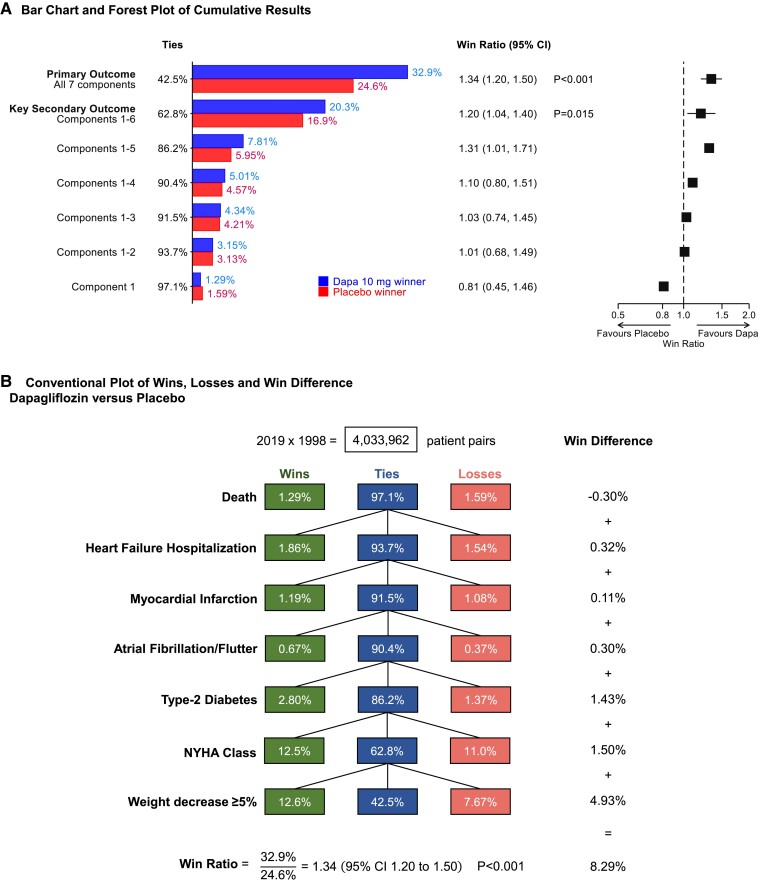
Two alternative ways of plotting win ratio findings from the DAPA-MI trial.^38^ (*A*), from the trial publication, shows the cumulative results over the hierarchy, but does not explicitly show the percentage of ties. (*B*), adopting the style most commonly used, shows the percentage of wins, losses, and ties at each level of the hierarchy. The right column helps clarify what is happening by showing the win difference at each level. DAPA, dapagliflozin; CI, confidence interval; NYHA, New York Heart Association

*Figure 5* shows two alternative ways of displaying these win ratio findings. The first, from the trial publication, concentrates on cumulative results over the hierarchy. The second is in the same style as other win ratio plots. In particular, the win difference column helps clarify what is happening. The first four components make little contribution, with win differences ± .3% or less. Modest treatment benefits for type 2 diabetes incidence and improved NYHA class contribute win differences around 1.5%, but much the biggest contribution is weight loss with win difference close to 5%.

One feels this elaborate win ratio analysis did not capture a meaningful benefit of dapagliflozin. Its effects on weight loss^39^ and avoiding diabetes^40^ are already known, change in NYHA class is a ‘soft’ outcome, and evidence on reducing CV events or deaths is lacking.

#### *Post hoc* exploratory win ratio analysis

When a trial fails to show benefit for its primary endpoint, exploratory analyses are often pursued, seeking more positive findings.^41^  *Post hoc* win ratio analyses are an option.

**Figure 6:**
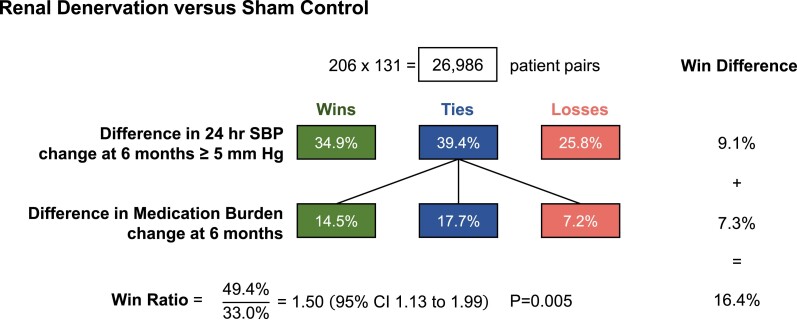
An example of a *post hoc* win ratio analysis of 24-h ambulatory systolic blood pressure and medication burden in the SPYRAL HTN-ON MED trial.^42^ The primary endpoint, mean change in ambulatory systolic blood pressure over 6 months, did not reach statistical significance. Increases in medication intensity among sham controls were substantial, diluting a true effect. Hence, this *post hoc* win ratio analysis has hierarchy (i) change in ambulatory systolic blood pressure at 6 months, with a margin of 5 mmHg, and (ii) change in medication burden at 6 months, with no margin. SBP, systolic blood pressure; CI, confidence interval; hr, hour

One example is the SPYRAL HTN-ON MED trial^42^ of renal denervation vs. sham control in 337 patients with resistant hypertension on anti-hypertensive medication. The primary endpoint, mean change in ambulatory systolic blood pressure (SBP) over 6 months, had a treatment difference of −1.9 mmHg (95% CI −4.4, .5; *P* = .12). Increases in medication intensity among sham controls were substantial, and hence may have diluted the true effect of renal denervation. Hence, a *post hoc* win ratio analysis was undertaken with hierarchy (i) change in ambulatory SBP at 6 months, with a margin of 5 mmHg, and (ii) change in medication burden at 6 months, with no margin. The win ratio is 1.50 (95% CI 1.13–1.99; *P* = .005), with both differences in SBP and medication burden contributing win differences 9.1% and 7.3%, respectively (*Figure 6*).

While such *post hoc* analyses are interesting, they do not directly influence a trial’s conclusions. This example illustrates how a clinical hierarchy of endpoints and its win ratio can be useful in trials where deaths and clinical events are not relevant.

Many trials have published *post hoc* win ratio analyses when the original primary endpoint is a conventional composite.^3^ If every component of the composite shows treatment benefit, e.g. COMPASS,^43^ then inevitably, analyses using win ratio and hazard ratio will be consistent. But if treatment benefit is confined to events lower in the hierarchy with no influence on mortality, then win ratio analysis will rightly show a lesser effect.

### Stratified win ratio, matched win ratio, and subgroup analysis

Many trials have stratified randomization. It is then appropriate to stratify analyses, either based on the same factors or others of key interest. We explain how this works for win ratio analyses.

**Figure 7:**
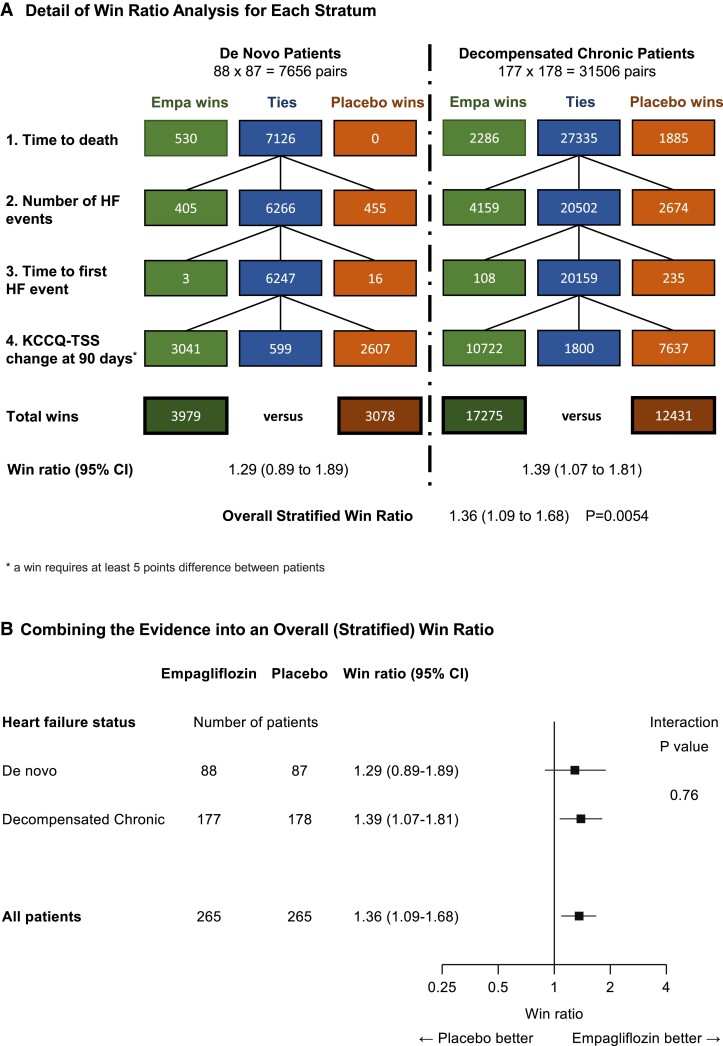
The stratified win ratio in the EMPULSE trial^26,27^: combining win ratio estimates for the two strata into an overall estimate. (*A*) shows details of the win ratio analysis for each stratum (*de novo* and decompensated chronic patients) with stratum-specific win ratios and an overall stratified win ratio. (*B*) shows the stratum-specific win ratios with 95% confidence intervals and an interaction *P*-value, plus the overall stratified win ratio with 95% confidence interval. Empa, empagliflozin; HF, heart failure; KCCQ-TSS, Kansas City Cardiomyopathy Questionnaire-Total Symptom Score; CI, confidence interval

The EMPULSE trial’s^26,27^ unstratified analysis is already presented in *Figure 4A*. The pre-defined primary analysis was stratified by *de novo* acute HF and decompensated chronic HF (*Figure 7*). The two subgroup win ratios 1.29 and 1.39, respectively (*Figure 7A*), are plotted with CIs in *Figure 7B*. An overall stratified win ratio CI and *P*-value were then obtained (see Supplementary data online, *Appendix*, for details).

Note that the stratified and unstratified win ratios are very similar. We expect this, unless the strata have markedly different risk profiles across the hierarchy. A quick check is that the two strata’s % ties 7.6% and 5.7% are similar.

Lastly, an interaction test comparing the stratum specific win ratios yields interaction *P* = .76 for EMPULSE. Such subgroup analysis interaction tests can be done comparing the log (win ratios) and their standard errors (see Supplementary data online, *Appendix*).

In general, stratifying a win ratio analysis leads to a slight loss of statistical power if the strata are of equal risk. On the other hand, if one or two patient factors strongly relate to patient risk, then stratified analysis will slightly enhance statistical power. Further examples and insights concerning the stratified win ratio are in the Supplementary data online, *Appendix*.

The RELIEVE-HF trial^25^ randomized 508 patients to interatrial shunt or sham control and has interesting subgroup analyses for the win ratio. The primary endpoint over 12–24 months was a hierarchical composite of death, cardiac transplant or left ventricular assist device (LVAD), all HF hospitalizations, all outpatient HF events, and change in KCCQ-OS at 12 months (with 5-point margin). The overall win ratio showed no effect, but subgroup analyses by left ventricular ejection fraction (LVEF) ≤40% or >40% showed evidence of heterogeneity (*Table 5*). For LVEF >40% (302 patients), there was evidence of harm: win ratio .70 (95% CI .54, .92), whereas for LVEF ≤40% (206 patients), there is a suggestion of benefit: win ratio 1.40 (95% CI .80, 2.46); interaction *P* = .0275.

**Table 5:** Subgroup analyses in the RELIEVE-HF trial^25^ by left ventricular ejection fraction for the primary win ratio and a composite of clinical events

Win ratio analysis								
	LVEF ≤40%				LVEF >40%			
	Shunt *N* = 101	Patient pairs 10 606	Placebo *N* = 106		Shunt *N* = 149	Patient pairs 22 797	Placebo *N* = 153	
	Wins	Ties	Losses	Win difference	Wins	Ties	Losses	Win difference
Death	14.6%	74.6%	10.9%	+3.7%	4.0%	83.4%	12.6%	−8.6%
CT or LVAD	4.4%	69.6%	.6%	+3.8%	0	83.4%	0	0
HF hospitalization	15.8%	40.6%	13.3%	+2.5%	13.5%	51.0%	18.9%	−5.5%
Outpatient HF events	4.8%	30.4%	5.4%	−.6%	7.0%	37.8%	6.2%	+.8%
Change in KCCQ	12.6%	4.7%	13.1%	−.5%	14.2%	6.2%	17.4%	−3.2%
Overall	52.1%		43.2%	+8.9%	38.6%		55.1%	−16.5%
	**Win ratio (95% CI)**							
Overall	.89 (.72, 1.09)							
LVEF ≤40%	1.21 (.87, 1.67)							
LVEF >40%	.70 (.54, .92)	Interaction *P* = .0275						

Rate ratio analysis of clinical events								
	LVEF ≤40%		LVEF >40%					
	Shunt *N* = 101	Placebo *N* = 106	Shunt *N* = 149	Placebo *N* = 153				
Death	13	20	22	7				
CT or LVAD	1	6	0	0				
HF hospitalization	41	78	87	47				
Outpatients’ HF events	21	30	34	34				
Total	76	134	143	88				
	**Rate ratio (95% CI)**							
LVEF ≤40%	.55 (.42, .73)							
LVEF >40%	1.68 (1.29, 2.19)	Interaction *P* < .0001						

in the primary publication, the win ratio was weighted before and after an adaptive design interim analysis and thus the results are slightly different. LVEF, left ventricular ejection fraction; CI, confidence interval; CT, cardiac transplant; LVAD, left ventricular assist device; HF, heart failure; KCCQ-TSS, Kansas City Cardiomyopathy Questionnaire.

Secondary analyses revealed no effect on KCCQ-OS, so removing this from the hierarchy enhanced the interaction to *P* = .01. However, the most striking evidence of interaction was a conventional (non-hierarchical) analysis of all the clinical events. For LVEF >40% and LVEF ≤40%, the consequent rate ratios were 1.68 (95% CI 1.29, 2.19) and .55 (95% CI .42, .73), respectively (interaction *P* < .0001).

While such subgroup findings need cautious interpretation, it appears the overall win ratio was ill-suited to capture this diversity of effects across subgroups and components of the primary outcome.

Lastly, the matched win ratio and covariate adjustment for the win ratio are topics of methodological interest, but as yet rarely used in practice (see Supplementary data online, *Appendix*, for details).

### Statistical software for win ratio analyses

Statistical software packages for analysing data using the win ratio are available in R and Stata. Several packages allow a flexible, user-defined hierarchy and include analysis of quantitative, binary, and time-to-event outcomes. Some packages offer analysis of repeat events, stratified analyses, or use of a margin for quantitative outcomes. Results obtained from software packages may differ due to how they handle missing data for quantitative outcomes. Therefore, the approach to missing data needs pre-specifying and an appropriate software package chosen (see Supplementary data online, *Appendix*, for details).

### Determining trial size for win ratio primary outcome

Trials based on the win ratio require a means of determining trial size. The principles behind such power calculations are unchanged, but often require simulations for the win ratio. The details of this methodology and its statistical software are in the Supplementary data online, *Appendix*.

Adaptive sample size re-estimation is often advocated for trials. This can best be done for a win ratio outcome when the interim analysis contains complete primary outcome data for each patient, using the promising zone methodology^44^ (see Supplementary data online, *Appendix*, for details).

The win ratio approach can be adapted to non-inferiority trials, a key step being to define an appropriate non-inferiority margin for the win ratio^45^ and a consequent justification of trial size. We intend to document this in a subsequent publication.

### Limitations

Some of our attempts to provide guidance on various aspects of the win ratio approach are based on experience from specific examples, so some caution is warranted until they are backed up by further investigation, including simulation studies. Our claim about increased use of the win ratio is based on anecdotal evidence but will be supported by a systematic review of published trials with the win ratio that is currently in progress.

## Conclusions

The value of hierarchical composite outcomes and the win ratio in cardiology trials is being increasingly recognized. This article has elucidated the diversity of its application, including hierarchies of death and clinical events, repeat events, and quantitative outcomes. The consequent statistical developments are also documented with added detail in the Supplementary data online, *Appendix*. Lessons learnt regarding potential misuses of the win ratio are also tackled. In order to aid wise future use of the win ratio, the *Graphical Abstract* provides a set of recommendations.

## Supplementary data

Supplementary data are available at *European Heart Journal* online.

## Supplementary Material

ehae647_Supplementary_Data

## Data Availability

No data were generated or analysed for this manuscript.
