# Supplementary material for: The win ratio in cardiology trials: lessons learnt, new developments, and wise future use
Source: Eur Heart J. 2024 Oct 15;45(44):4684–99. doi: 10.1093/eurheartj/ehae647 (PMC11578645; doi:10.1093/eurheartj/ehae647)
Supplement: ehae647_Supplementary_Data [file ehae647_supplementary_data.pdf]

STATISTICAL APPENDIX

Contents

Finkelstein-Schoenfeld test 2
Win ratio and its 95% confidence interval 3
    Unmatched win ratio 3
    Matched win ratio 3
Win difference, win odds and their 95% confidence intervals 5
Stratified win ratio and its 95% confidence interval 5
    A simple pooled approach 5
    Inverse-variance approach 6
    Patient-weighted approach 7
    Mantel-Haenszel type weighting 7
Further Examples of The Stratified Win Ratio 8
Subgroup Analyses and Calculating Interaction P-value 11
Matched Win Ratio and Covariate-Adjusted Win Ratio 11
Statistical software for the win ratio 12
Determining Trial Size for Win Ratio Primary Outcome 15
Adaptive Sample Size Re-Estimation 17
References 20

Finkelstein-Schoenfeld test

The method for determining the p-value for the unmatched win ratio was introduced by Finkelstein and Schoenfeld (1). Suppose there are Nt, Nc patients on treatment and control respectively, so total sample size $N = Nt + Nc$. The process for calculating a p-value proceeds as follows:

1) **Irrespective of treatment group** we compare all possible pairs of patients *i, j* to determine whether patient *i* was the winner, the loser or they tied, making a total of N^2 comparisons.
2) For each pair of patients i and j we create $U_{ij} = 1$ if patient *i* was the winner, $U_{ij} = -1$ if patient *i* lost, or $U_{ij} = 0$ if they tied.
3) For patient *i* we define $U_i = \sum_{i \neq j} u_{ij}$. Note U_i will be positive if patient *i* wins more often than he loses compared to all other patients and negative if patient *i* loses more often than he wins.
4) Then we calculate $T = \sum_{i=1}^{N} U_i D_i$ where $D_i = 1$ if patient *i* is on the new treatment and $D_i = 0$ if patient *i* is on standard treatment.
5) Under the null hypothesis of no true difference between new and standard treatment, *T* has mean 0 and variance *V* where $V = \frac{N_c N_t}{N(N-1)} \sum_{i=1}^{n} U_i^2$.
6) $Z = \frac{T}{\sqrt{V}}$ is a standardised normal deviate from which a p-value can be readily obtained. Specifically, Z >1.96, Z >2.38 and Z > 3.29 correspond to P<.05, P<.01 and P<.001 respectively.

Win ratio and its 95% confidence interval

Unmatched win ratio

To calculate the win ratio we compare all treated patients to all control patients, making a total of $Nt \times Nc$ comparisons. We calculate the number of wins and losses. The win ratio is then estimated as the total number of wins (N_W) divided by the total number of losses (N_L).

Under the null hypothesis the log win ratio is approximately normally distributed and its standard error can be estimated by $SE_{logWR} = \frac{\log\left(\frac{N_W}{N_L}\right)}{Z}$, where Z is the Z-statistic obtained from the Finkelstein-Schoenfeld test, as described in the previous section. A 95% confidence interval can then be calculated for the log win ratio and this can be back-transformed to give a 95% confidence interval for the win ratio, i.e.

$$\exp\left(\log\left(\frac{N_W}{N_L}\right) - 1.96\ \times SE_{logWR}\right),\quad \exp\ (\log\left(\frac{N_W}{N_L}\right) + 1.96\ \times SE_{logWR})$$

An alternative is to use the variance estimator of Dong et al (2) to calculate the standard error of the log win ratio, although the two approaches tend to give almost identical results except when the observed win ratio is very far from 1.

Matched win ratio

To perform a matched win ratio one first needs to define an algorithm for matching patients from the intervention arm to the control arm. One could use a range of methods for this purpose, such as those developed for matching based on the propensity score. In the EMPEROR studies we have used the following simple procedure to match patients:

1) For 1:1 matching we require that the treatment arms be of equal size. We therefore began the process by randomly dropping a small number of patients from the arm with the greater number of patients. This meant that 4 randomly chosen placebo patients in EMPEROR-Reduced and 6 randomly chosen empagliflozin patients in EMPEROR-Preserved were removed from the analysis. Note that instead of randomly dropping patients one could use a more sophisticated algorithm, for example selecting patients for removal in

order to minimize the between group difference in mean predicted risk (as calculated in Step 2).

2) For the remaining patients we calculated their risk score on the basis of the covariates in the EMPEROR risk scores (3, 4). (after excluding randomised treatment from each model).
3) Within treatment group, patients were ordered from lowest risk to highest risk. If there was a tie (same value of risk score) then patients were ordered according to date of randomisation
4) The lowest risk patient in the empagliflozin group was matched to the lowest risk patient in the placebo group, the second lowest-risk patient in the empagliflozin group was matched to the second-lowest risk patient in the placebo group and so on.

Within each pair of patients, we evaluated whether it was a ‘win’, ‘tie’ or ‘loss’. The matched win ratio was then calculated as follows:

1) Calculate the total number of wins N_w and losses N_L
2) Calculate the estimated win ratio as $R_w = \frac{N_w}{N_L}$
3) Calculate the proportion of wins amongst untied patients $p_w = \frac{N_w}{N_w + N_L}$
4) p_w is a proportion and so the confidence limits for p_w are found at $p_w \pm 1.96\sqrt{\frac{p_w(1-p_w)}{N_w + N_L}} = p_{lower}, p_{upper}$
5) The win ratio = $\frac{p_w}{1-p_w}$ and so a 95% confidence limit for the win ratio can be found at $\frac{p_{lower}}{1-p_{lower}}, \frac{p_{upper}}{1-p_{upper}}$
6) The Z-statistic can be calculated using $Z = \frac{(p_w - 0.5)}{\sqrt{\frac{p_w(1-p_w)}{N_w + N_L}}}$. This is a standard normal deviate under the null hypothesis readily yielding a p-value.

Win difference, win odds and their 95% confidence intervals

The win difference is calculated as: $D_w = 100 \times (\frac{N_W - N_L}{NtNc})$

We note that the numerator of the win difference $(N_W - N_L)$ is equal to the numerator in the Finkelstein Schoenfeld test defined on page 2 of this Appendix ($T = \sum_{i=1}^{N} U_i D_i$).

The win odds is calculated as $O_w = \frac{N_W + 0.5(NtNc - N_W - N_L)}{N_L + 0.5(NtNc - N_W - N_L)}$

The win difference and the win odds have the same null hypothesis as for the win ratio and hence the p-values and Z-statistics described above for the win ratio remain valid.

Under the null the win difference is asymptotically normally distributed with mean 0, and hence its standard error can be estimated by (the absolute value of) $\frac{D_w}{Z}$, which can then be used to calculate a 95% confidence interval.

The log win odds, like the log win ratio is expected to be approximately normally distributed with mean 0. Its standard error can be estimated by $\frac{\log(O_W)}{Z}$.

Alternative variance estimators give similar results.

Stratified win ratio and its 95% confidence interval

There are several ways to calculate a stratified win ratio. But all methods work by **only comparing patients who are in the same stratum** (comparing all possible pairs of patients within a stratum) and then combining results across strata. In what follows we assume that there are K such strata.

A simple pooled approach

Within each stratum, k, one calculates the number of wins $n_{w,k}$ and number of losses $n_{l,k}$. Then the overall win ratio is then calculated as

$$R_w = \frac{\sum_{k=1}^{K} n_{w,k}}{\sum_{k=1}^{K} n_{l,k}}$$

Within each stratum the test statistic T_k and its variance V_k are calculated as for the unmatched win ratio. These are then pooled across strata as follows:

$$Z = \frac{\sum_{k=1}^{K} T_k}{\sqrt{\sum_{k=1}^{K} V_k}}$$

Calculation of p-values and standard errors then proceeds in the same way as for the unmatched win ratio.

A major limitation of this approach is that it over-emphasizes the results from large strata, as illustrated by the following hypothetical example. Suppose we run a trial with 100 patients in each treatment group. Suppose there are 10 patients in each of the intervention and control group in Stratum 1 and 90 in Stratum 2. Then Stratum 1 yields 10×10 =100 pairwise comparisons and Stratum 2 yields 90×90 =8100. Therefore, if the event rate in the two Strata is identical, we expect that the relative contribution of Stratum 2 to Stratum 1 will be $\frac{8100}{100}$=81 times greater, despite only containing 10 times as many patients.

Inverse-variance approach

1) Within each stratum k, one calculates the number of wins $n_{w,k}$ and number of losses $n_{l,k}$
2) The stratum-specific log win ratio $\log(R_{w,k})$ and its standard error SE_k are then calculated separately within each stratum using the same methodology as using an unmatched approach. is calculated
3) Weights are calculated as the inverse of the variance of the log win ratio normalized so that the sum of weights sums to 1:

$$\text{weight}_k = \frac{\frac{1}{{SE_k}^2}}{\sum_{k=1}^{K} \frac{1}{{SE_k}^2}}$$

4) The log win ratio and it's standard error are then calculated as follows:

$$\log(R_w) = \sum_{k=1}^{K} \text{weight}_k \log(R_{w,k})$$

$$SE = \sqrt{\sum_{k=1}^{K} SE_k^2 \, weight_k^2}$$

Patient-weighted approach

The process is defined in the same was as for the inverse-variance approach above except that for each stratum the stratum-specific weights are defined as $\mathrm{weight}_k = \frac{N_k}{\sum_{k=1}^{K} N_k}$ where N_k is equal to the total number of patients in stratum k (intervention and control). Groups are therefore weighted according to the number of patients.

Mantel-Haenszel type weighting

Within each stratum k, one calculates the number of wins $n_{w,k}$ and number of losses $n_{l,k}$. Let N_k be equal to the total number of patients in stratum k (intervention and control). Then the Mantel-Haenszel type weighted stratified win ratio refers to a win ratio calculated as follows:

$$R_w = \frac{\sum_{k=1}^{K} {n_{w,k}}/{N_k}}{\sum_{k=1}^{K} {n_{l,k}}/{N_k}}$$

The standard error of the log win ratio is given by:

$$SE = \sqrt{\frac{\sum_{k=1}^{K} {\widehat{\sigma_{t,k}^2}}/{N_k}^2 + \sum_{k=1}^{K} {\widehat{\sigma_{c,k}^2}}/{N_k}^2 + 2\sum_{k=1}^{K} {\widehat{\sigma_{tc,k}}}/{N_k}^2}{(\frac{1}{2}\sum_{k=1}^{K} {n_{w,k}}/{N_k} + \sum_{k=1}^{K} {n_{l,k}}/{N_k})^2}}$$

Where $\widehat{\sigma_{t,k}^2}$, $\widehat{\sigma_{c,k}^2}$ refer to the estimated variance of the within-stratum number of wins and losses respectively and $\widehat{\sigma_{tc,k}}$ refers to their covariance. Formulae for $\widehat{\sigma_t^2}$, $\widehat{\sigma_c^2}$, $\widehat{\sigma_{tc}}$ are given in Dong (2). This weighting scheme is termed Mantel-Haenszel type because in the case of a binary outcome, a win ratio weighted in this way is identical to the inverse of the Mantel-Haenszel odds ratio (5).

Further Examples of The Stratified Win Ratio

The ATTR-ACT trial (6, 7) is an interesting example of a stratified win ratio analysis. The trial randomized 441 patients with transthyretin amyloid cardiomyopathy in a 2:1:2 ratio to 8 mg tafamidis, 20 mg tafamidis or placebo. The primary analysis over 30 months compared both doses combined versus placebo for the hierarchical composite endpoint of 1) all-cause death and 2) number of CV hospitalisations (CVHs). Analysis was stratified by NYHA class (I/II or III) and TTR status (variant or wild type) leading to separate analyses for 4 strata (Figure S1). The overall stratified win ratio is 1.70 (95% CI 1.26-2.29) P=0.0006 which is an elegant way of combining the impact of tafamidis on both mortality and CVH into a highly positive conclusion.

But two details are worth noting. First, patient risk varied markedly across the 4 strata, the % ties being 23.6%, 16.7%, 7.6% and 2.5% indicating that both NYHA class III and variant type contributed to a worse prognosis. This supports the use of a stratified analysis. Secondly, the stratum-specific win ratios varied markedly 1.92, 1.55, 1.07 and 0.86 which is suggestive of potential heterogeneity of treatment effect. Appropriate subgroup analyses and statistical tests of interaction would have been helpful. But ATTR-ACT pioneered the use of win ratio, with its design in 2013 and publication in 2018, when such win ratio methodology was still in development.

The ATTRibute trial (8)(Figure 4C in main article) is another example of stratified win ratio analysis. There were originally 2 x 2 x 2 = 8 strata according to TTR genotype (wild-type or variant), NT proBNP (>3000 pg/ml or less), eGFR (<45 ml/min/1.73 m^2 or more). But some strata contained too few patients for analysis and hence the primary analysis collapsed then into 5 strata in all. This indicates a warning that one should avoid proposing too many strata in a win ratio analysis.

Figure S1: Stratified Win Ratio Analysis of the ATTR-ACT Trial (6,7): the hierarchical composite is death then number of cardiovascular hospitalizations (CVHs) and the 4 strata based on NYHA class (I/II or III) and TTR status (wild or variant type)

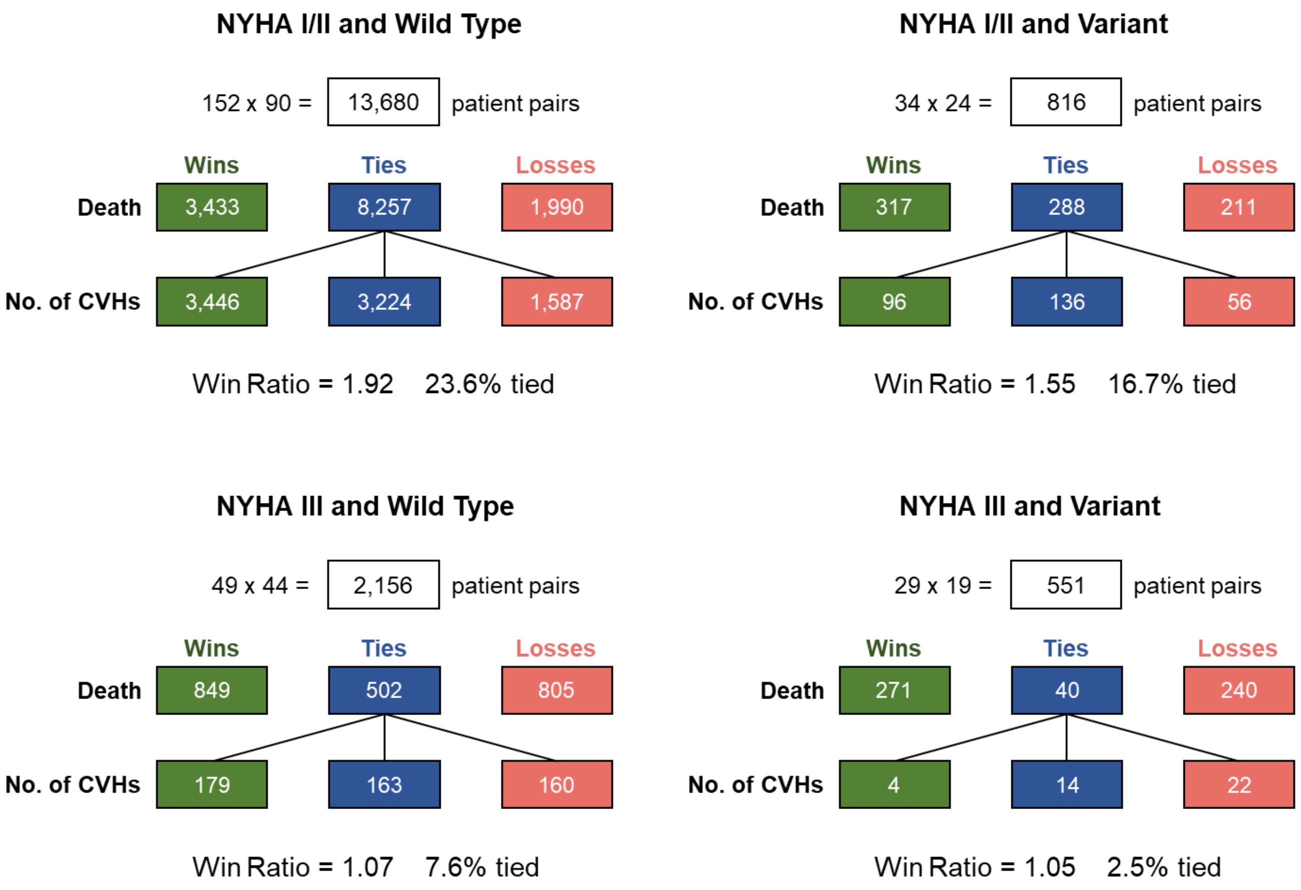

Stratified Win Ratio Analysis of the ATTR-ACT Trial (6,7). The hierarchical composite is death then number of cardiovascular hospitalizations (CVHs) and the four strata based on NYHA class (I/II or III) and TTR status (wild or variant type). Details of the number of wins, losses and ties are shown for each level of the hierarchy within each of the four stratum, along with the stratum specific win ratio and an overall stratified win ratio, 95% CI and p-value. NYHA, New York Heart Association; TTR, Transthyretin; CVH, cardiovascular hospitalizations; CI, confidence interval.

Risk stratification can reveal interesting win ratio results. For EMPEROR Preserved the key overall results for the hierarchical composite of CV death and HF hospitalisation have already been presented in Figure 3C in the main paper. Of relevance is the development of a risk score for the trial's primary outcome time to CV death or HF hospitalisation, which facilities classification of individual patients into equal-sized groups of low, medium and high-risk (4). The consequent risk-

stratified win ratio analysis findings are in Figure S2. The win ratio is similar across the three strata (1.18, 1.37 and 1.24 respectively) though the 95% CI gets substantially narrower with increased risk due to the greater frequency of primary events. This is reflected in the % ties by risk stratum: 91.7%, 81.0% and 56.9% respectively. The win difference by risk stratum varies markedly: 0.7%, 2.9% and 4.6% respectively for low-, medium- and high-risk patients (P for trend =0.026) This clearly indicates that the absolute benefit of empagliflozin versus placebo is more marked in higher risk patients.

Note the overall risk-stratified win ratio is 1.27 (P=0.0006) slightly greater than the unstratified 1.25 (P=0.012). Repeating this exercise with 5 or 10 equal-sized strata gave comparable results with no further gain in statistical power.

Figure S2. A Risk Stratified Win Ratio Analysis for the Hierarchical Composite Endpoint CV death and HF Hospitalization in the EMPEROR Preserved Trial (9)

Risk Group	N empa	N placebo	N of pairs	wins	ties	losses	Win Ratio (95% CI)	Win Difference (95% CI)
Low	1019	972	995,563	4.5%	91.7%	3.8%		
Medium	964	1032	994,848	11.0%	81.0%	8.0%		
High	1014	982	995,748	23.9%	56.9%	19.2%		

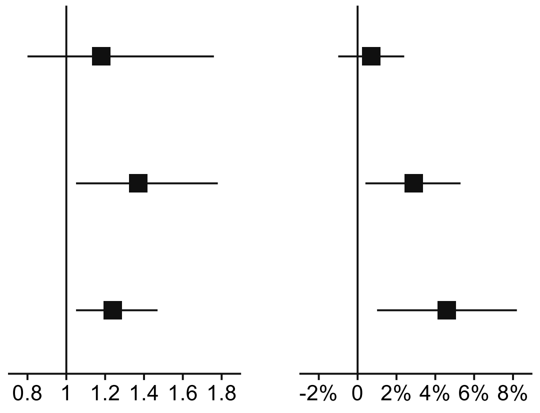

A Risk Stratified Win Ratio Analysis for the Hierarchical Composite Endpoint CV death and HF Hospitalization in the EMPEROR Preserved Trial (9). Details of the win ratio analysis by low, medium and high risk group. Win ratio and win differences for each stratum are presented along with 95% CIs. CV, cardiovascular; N, number; CI, confidence interval.

Subgroup Analyses and Calculating Interaction P-value

The above sections describe how to calculate the estimated log win ratio $\log(R_w)$ and its standard error SE_{logWR}.

To test for a difference between two strata or subgroups one can use the following procedure. Suppose we have win ratio in groups 1 and 2 $\log(R_{w1})$, $\log(R_{w2})$ respectively and their corresponding standard errors SE_{logWR1}, SE_{logWR2}. Then a Z-statistic testing for interaction can be calculated as:

$$\frac{\log(R_{w1}) - \log(R_{w2})}{\sqrt{{SE_{logWR1}}^2 + {SE_{logWR2}}^2}}$$

and a p-value can be calculated by comparing the Z-statistic to a standard normal distribution in the usual way.

If there are more than 2 strata and they are ordered, then one may wish to instead test for a linear trend in the log win ratio across ordered categories. To do so one can use a variance weighted least squares regression, where the within-stratum log win ratio is the dependent ("y") variable, the ordered values of the strata is the independent ("x") variable and the within-stratum estimated variance of the log win ratio (i.e. the within-stratum standard error squared) is used to weight the regression.

If one instead wishes to calculate tests of interaction for the win difference, then one substitutes into the above procedures as follows:

(i) the estimated win difference is used in place of the estimated log win ratio

(ii) the estimated standard error of the win difference is used in place of the estimated standard error of the log win ratio.

Matched Win Ratio and Covariate-Adjusted Win Ratio

The original win ratio article in 2012 (10) also advocated use of the matched win ratio, which attempts to account for each patient's underlying risk by forming matched pairs of patients from the treatment and control groups. It was argued that it could increase the magnitude of the win ratio and hence increase statistical power.

But in examples we have tried, EMPEROR Preserved and Reduced, the confidence interval width was greater compared with the unmatched win ratio. The matched approach often loses some patients from analysis who could not be matched, and it is also difficult to pre-specify the matching process. Hence, we do not recommend its use for randomised trials. However, in non-randomized registry studies with a much larger control group available, one could consider matching on a propensity score as a viable route to a win ratio analysis (11).

One concept needing further development is covariate-adjustment for the win ratio. For a conventional composite endpoint it is straightforward to add covariates to the Cox proportional hazard model, and if such covariates are strongly associated with patient risk then the estimated treatment effect may be enhanced (12). For instance, for the primary composite endpoint of EMPEROR-Preserved, the unadjusted result (Table 1 in main paper) was hazard ratio 0.79 (95% CI 0.69-0.90). After allowance for 8 covariates strongly related to patient prognosis, the adjusted hazard ratio became 0.75 (95% CI 0.66-0.86). At present no equivalent method exists for a covariate-adjusted win ratio. However, Wang et al (13) adopt an alternative approach which corrects for baseline imbalance in covariates: the adjusted win ratio uses inverse probability of treatment weighting. They demonstrate desirable statistical properties, but the influence of covariates on patient risk is not directly considered.

Another related issue is to adjust for the baseline value when analysing the change in a quantitative component of the win ratio, such as KCCQ score. The logic is that patients with a higher baseline score have less scope to improve, ie change and baseline value are negatively correlated, and this fact is ignored in the crude current methods. A solution is to replace a patient’s change in score by the residual from the regression line of change versus baseline in all patients. This should slightly enhance statistical power.

Statistical software for the win ratio

Statistical software packages available for analyzing data using the win ratio approach are available in R (14-16) and Stata (17, 18) [Table S1]. Several packages

now allow a flexible, user-defined hierarchy and include the analysis of quantitative, binary or time-to-event outcomes. Some packages offer additional features such as analysis of recurrent events, stratified analyses, or the specification of a minimally clinically important difference (margin) in the analysis of quantitative outcomes. Where quantitative or binary covariates form part of the hierarchy results obtained from software packages may differ due to their approach to handling missing data. Therefore the approach to handling missing data should be pre-specified and an appropriate software package chosen.

Table S1: Summary of publicly available statistical software for analysing data using the win ratio

Platform	Package and installation	Outcome types	Approach to missing data for continuous or binary outcomes*	Variance estimator used**	Stratified win ratio	Permits use of a margin in the analysis of continuous outcomes	Reports other statistics, e.g. win odds and win difference
Stata	winratiotest available from the Stata SSC archive. Type “net install winratiotest”	Binary, ordinal continuous, time-to-event, repeat events	Considered a tie	Null variance	Yes	Yes	No
R	BuyseTest (available from the Comprehensive R network: CRAN)	Binary, ordinal continuous, time-to-event	Various options	Null variance, or estimated via bootstrap	Yes	Yes	Yes
	WinRatio (available from the Comprehensive R network: CRAN)	Binary, ordinal continuous, time-to-event, repeat events	Case-wise deletion	Asymptotic	No	No	No
	WINS (available from the Comprehensive R network: CRAN)	Binary, ordinal, continuous, time-to-event	Case-wise deletion	Null or asymptotic	Yes	Yes	Yes
SAS	To our knowledge there is no general publicly available software for SAS						

*For each patient pair, all outcomes which are higher priority than the outcome with missing data are evaluated. If a decision cannot be made on these outcomes and at least one of the pair has missing data, then the patient pair is considered a tie i.e. lower priority outcomes are not considered

**Null variances uses the method described in the Statistical Appendix. Asymptotic variance uses the variance estimator of Dong (5)

Determining Trial Size for Win Ratio Primary Outcome

Determining the required sample size is an important part of trial planning. Sample size calculations for trials using the win ratio have often required complex and time consuming simulations. Recently, a sample size formula for the win ratio was published (19), but the required inputs are the anticipated win ratio and proportion of paired comparisons that are tied under the alternative hypothesis. These concepts are abstract and often not obvious from previous trials.

To aid sample size determination for the win ratio we have developed free publicly-available statistical software in Stata (17) for sample size calculations that can take as inputs the anticipated distribution of each component of the win ratio hierarchy in more conventional terms, such as hazard ratios, proportion of patients with an event or differences in means. The software can combine any number of time-to-event, binary, repeat or continuous outcomes in a hierarchy in any order. The software makes two simplifying assumptions: (1) that follow-up duration is uniform; (2) that component outcomes at each level of the hierarchy are independent from one another. Although these assumptions are sometimes unrealistic, the impact of modest deviations from these assumptions on trial size is often small. It can therefore be useful to calculate approximate sample sizes using our software during before the study design is finalized in order to explore possible scenarios. More exact sample size calculations undertaken by simulation may then be helpful once the study design is settled.

We consider how this could have been done in two trials that used the win ratio for the primary analysis: ATTRIBUTE-CM (8) and EMPULSE (20). In ATTRIBUTE-CM the primary outcome used a hierarchy of (1) time-to-death, expected to occur in 40% of placebo patients with an anticipated hazard ratio for acoramidis of 0.7; (2) number of CV hospitalisations with an expected mean number of hospitalisations of 0.75 in the acoramidis and 1.15 in the placebo arm. There was a 2:1 randomisation ratio. A type I error rate of 0.04 was planned, lower than the conventional 0.05 to allow for a pre-planned interim analysis. The authors reported that simulations showed that 460 patients gave >90% statistical power.

We replicate this with the following statistical code in Stata, which shows that 460 patients should provide approximately 92% power under these assumptions.

```
winratiopower, alpha(0.04) nratio(2) n(460) outcome(tf eventprob(0.4) hr(0.7))
        outcome(r mean(0.75 1.15) win(fewer))

Estimated sample size for the win ratio

Study parameters:
  alpha = 0.04
  N = 460

 Estimated percentage of wins, losses and ties:
  ------------------------------------------
  Level       Wins    Ties    Losses
  ------------------------------------------
  Level 1     0.34    0.42    0.24
  Level 2     0.19    0.13    0.10
  ------------------------------------------
  Overall     0.53    0.13    0.34
  ------------------------------------------

 Estimated power: 0.92
```

The EMPULSE trial was designed to achieve 87.4% power at a type I error rate of 5% using a hierarchical outcome consisting of: 1) time-to-death, expected to occur in 5% of placebo patients with an expected hazard ratio of 0.8 for empagliflozin; 2) number of repeat heart failure hospitalisations (HFH) with a mean number of 0.15 and 0.105 expected to occur in the placebo arm and empagliflozin arms respectively and expected to cluster in high risk patients such that they would follow a negative binomial distribution; 3) time-to-first HFH; and 4) a 5-unit or greater difference in KCCQ score. KCCQ was expected to be normally distributed with means of 55 and 61 in the placebo and empagliflozin arm respectively. The standard deviation of KCCQ was expected to be 20 in both arms.

For the purposes of illustration we ignore the third component of the hierarchy (time to first HFH). It was strongly related to the second component (number of HFH), was only responsible for breaking <1% of ties and so had negligible impact on the required sample size. Given these features, the required sample size can be estimated using the following code (all one line):

```
winratiopower, power(0.874)  alpha(0.05) outcome(tf eventprob(0.05) hr(0.8))
        outcome(r mean(0.105 0.15) win(fewer) dispersion(1))
        outcome(c mean(61 55) sd(20 20) win(higher) margin(5))
```

```
Estimated sample size for the win ratio
Study parameters:
  alpha = 0.05
  power = 0.87

 Estimated percentage of wins, losses and ties:
  ------------------------------------------
  Level      Wins   Ties   Losses
  ------------------------------------------
  Level 1    0.05   0.91   0.04
  Level 2    0.11   0.73   0.08
  Level 3    0.37   0.10   0.25
  ------------------------------------------
  Overall    0.53   0.10   0.37
  ------------------------------------------
 Estimated sample size:
  N = 470.5
  N per group = 235.3
```

The estimated sample size is 471 patients. This is slightly smaller than the required sample size of 500 determined by the study statisticians using simulations which take account of more complex anticipated features of the trial data (e.g. dependency between time to death, rate of HFH and KCCQ). However, the estimate is close enough to give trialists and sponsors a reasonable idea of sample size to aid discussions around trial design. The final sample size then can be more precisely calculated via simulation.

Adaptive Sample Size Re-Estimation

Although most trials are designed with a fixed sample size, it is also possible to use an adaptive design whereby the sample size is re-estimated based on the unblinded results of an interim analysis. We focus here on the 'promising zone' methodology of Mehta and Pocock (21), which for a conventional outcome works as follows. At the interim analysis one estimates the treatment effect for the primary outcome and its associated Z-statistic. The following formula is used to calculate the conditional power, that is, the statistical power of the trial given the results at the interim analysis:

$$CP = 1 - \Phi\left[\frac{z_\alpha\sqrt{n_2} - z_1\sqrt{n_1}}{\sqrt{\tilde{n}_2}} - \frac{z_1\sqrt{\tilde{n}_2}}{\sqrt{n_1}}\right]$$

Where:

CP is the conditional power

n_1 is the sample size at the interim analysis

n_2 is the total sample size at the end of the study

$\tilde{n}_2 = n_2 - n_1$ is the difference between the total sample size and the interim sample size

z_1 is the Wald test statistics at the interim analysis

z_α is the required Z-statistic at the final analysis to declare study success (i.e. typically 1.96 for a 5% significance threshold)

Φ is the cumulative distribution function for a standard normal distribution

Depending on these results, the trial may adapt in one of several ways. When the results of the interim analysis are in the promising zone (typically when conditional power is >50% but less than the pre-planned statistical power), an updated sample size is calculated in order to achieve a certain conditional power using the formulae of Mehta and Pocock (21).

A key assumption underlying this methodology is that the results of the interim analysis and the final analysis must be independent of one another. With a win ratio outcome this means that we must restrict the interim analysis to patients who already have complete primary outcome data at the time of analysis. Including data from patients with incomplete follow-up risks inflating Type I error and may also result in inappropriate adaptive strategies. Restricting the interim analysis in this way works well when the outcome is short-term because the time between recruitment and outcome ascertainment is short. Therefore, few patients have incomplete follow-up and little information is lost by excluding their (partial) primary outcome data. But for trials with longer term outcomes many patients may have incomplete follow-up and ignoring information from such patients makes the adaptive design less efficient. Methods to incorporate data from patients with incomplete follow-up under an adaptive design using the win ratio are described by Schoenfeld (22), but are complex and require simulations to be performed at the interim analysis.

An important consideration is that if the effect of the intervention is not consistent across the components of the hierarchy then the win ratio estimated at the interim

analysis will systematically differ from the win ratio estimated at the end of the trial. Hence it will be a poor basis for sample size re-estimation. For example, in TRILUMINATE the 1-year benefit was driven almost entirely by KCCQ improvements with almost no effect on clinical events (death, tricuspid-valve surgery, or heart failure hospitalisations). An interim analysis including patients with incomplete follow-up would mean including information from patients yet to have the 1-year KCCQ measurement, and so incorporate only information on clinical events. The interim analysis would therefore tend to yield a win ratio closer to 1 than what was seen at the final analysis, since the latter would include more comparisons based on 1 year KCCQ. Such an overly-pessimistic interim estimate could lead to a poor decision regarding sample size adaptation.

References

1. Finkelstein DM, Schoenfeld DA. Combining mortality and longitudinal measures in clinical trials. Stat Med. 1999;18(11):1341-54.
2. Dong G, Li D, Ballerstedt S, Vandemeulebroecke M. A generalized analytic solution to the win ratio to analyze a composite endpoint considering the clinical importance order among components. Pharm Stat. 2016;15(5):430-7.
3. Pocock SJ, Ferreira JP, Gregson J, Anker SD, Butler J, Filippatos G, et al. Novel biomarker-driven prognostic models to predict morbidity and mortality in chronic heart failure: the EMPEROR-Reduced trial. Eur Heart J. 2021;42(43):4455-64.
4. Pocock SJ, Ferreira JP, Packer M, Zannad F, Filippatos G, Kondo T, et al. Biomarker-driven prognostic models in chronic heart failure with preserved ejection fraction: the EMPEROR-Preserved trial. Eur J Heart Fail. 2022;24(10):1869-78.
5. Dong G, Qiu J, Wang D, Vandemeulebroecke M. The stratified win ratio. J Biopharm Stat. 2018;28(4):778-96.
6. Maurer MS, Schwartz JH, Gundapaneni B, Elliott PM, Merlini G, Waddington-Cruz M, et al. Tafamidis Treatment for Patients with Transthyretin Amyloid Cardiomyopathy. N Engl J Med. 2018;379(11):1007-16.
7. Pocock SJ, Collier TJ. Statistical Appraisal of 6 Recent Clinical Trials in Cardiology: JACC State-of-the-Art Review. J Am Coll Cardiol. 2019;73(21):2740-55.
8. Gillmore JD, Judge DP, Cappelli F, Fontana M, Garcia-Pavia P, Gibbs S, et al. Efficacy and Safety of Acoramidis in Transthyretin Amyloid Cardiomyopathy. N Engl J Med. 2024;390(2):132-42.
9. Anker SD, Butler J, Filippatos G, Ferreira JP, Bocchi E, Böhm M, et al. Empagliflozin in Heart Failure with a Preserved Ejection Fraction. N Engl J Med. 2021;385(16):1451-61.
10. Pocock SJ, Ariti CA, Collier TJ, Wang D. The win ratio: a new approach to the analysis of composite endpoints in clinical trials based on clinical priorities. Eur Heart J. 2012;33(2):176-82.
11. Von Koch S, Koul S, Erlinge D, Mohammad M. Percutaneous coronary intervention and medical therapy versus medical therapy alone in chronic coronary syndrome: A hierarchical win-ratio analysis from SCAAR. Eur Heart J. 2023;44(Supplement_2).
12. Pirondini L, Gregson J, Owen R, Collier T, Pocock S. Covariate Adjustment in Cardiovascular Randomized Controlled Trials: Its Value, Current Practice, and Need for Improvement. JACC Heart Fail. 2022;10(5):297-305.
13. Wang D, Zheng S, Cui Y, He N, Chen T, Huang B. Adjusted win ratio using the inverse probability of treatment weighting. J Biopharm Stat. 2023:1-16.
14. Cui Y, Huang B. WINS: The R WINS Package, 2022 [Available from: https://CRAN.R-project.org/package=WINS
15. Duarte K. WinRatio — Win Ratio for Prioritized Outcomes and 95% Confidence Interval.
16. Ozenne B PJ. BuyseTest: Implementation of the Generalized Pairwise Comparisons, 2021.
17. Collier T, Gregson J. WINRATIOTEST: Stata module to calculate the unmatched Win Ratio for prioritised outcomes [Available from: https://ideas.repec.org/c/boc/bocode/s458984.html.

18. Gregson J, Ferreira JP, Collier T. winratiotest: A command for implementing the win ratio and stratified win ratio in Stata. The Stata Journal. 2023;23(3):835-50.
19. Yu RX, Ganju J. Sample size formula for a win ratio endpoint. Stat Med. 2022;41(6):950-63.
20. Pocock SJ, Ferreira JP, Collier TJ, Angermann CE, Biegus J, Collins SP, et al. The win ratio method in heart failure trials: lessons learnt from EMPULSE. Eur J Heart Fail. 2023;25(5):632-41.
21. Mehta CR, Pocock SJ. Adaptive increase in sample size when interim results are promising: a practical guide with examples. Stat Med. 2011;30(28):3267-84.
22. Schoenfeld DA, Ramchandani R, Finkelstein DM. Designing a longitudinal clinical trial based on a composite endpoint: Sample size, monitoring, and adaptation. Stat Med. 2022;41(24):4745-55.
